# Regulation of FGF10 Signaling in Development and Disease

**DOI:** 10.3389/fgene.2018.00500

**Published:** 2018-10-23

**Authors:** Joanne Watson, Chiara Francavilla

**Affiliations:** ^1^Division of Molecular and Cellular Function, School of Biological Sciences, Faculty of Biology Medicine and Health, The University of Manchester, Manchester, United Kingdom; ^2^Division of Evolution and Genomic Sciences, School of Biological Sciences, Faculty of Biology Medicine and Health, The University of Manchester, Manchester, United Kingdom

**Keywords:** fibroblast growth factor 10, FGF receptor, signaling, development, cancer, genetic disorders, mass spectrometry, quantitative proteomics

## Abstract

Fibroblast Growth Factor 10 (FGF10) is a multifunctional mesenchymal-epithelial signaling growth factor, which is essential for multi-organ development and tissue homeostasis in adults. Furthermore, FGF10 deregulation has been associated with human genetic disorders and certain forms of cancer. Upon binding to FGF receptors with heparan sulfate as co-factor, FGF10 activates several intracellular signaling cascades, resulting in cell proliferation, differentiation, and invasion. FGF10 activity is modulated not only by heparan sulfate proteoglycans in the extracellular matrix, but also by hormones and other soluble factors. Despite more than 20 years of research on FGF10 functions, context-dependent regulation of FGF10 signaling specificity remains poorly understood. Emerging modes of FGF10 signaling regulation will be described, focusing on the role of FGF10 trafficking and sub-cellular localization, heparan sulfate proteoglycans, and miRNAs. Systems biology approaches based on quantitative proteomics will be considered for globally investigating FGF10 signaling specificity. Finally, current gaps in our understanding of FGF10 functions, such as the relative contribution of receptor isoforms to signaling activation, will be discussed in the context of genetic disorders and tumorigenesis.

## Introduction

**FIGURE 1:**
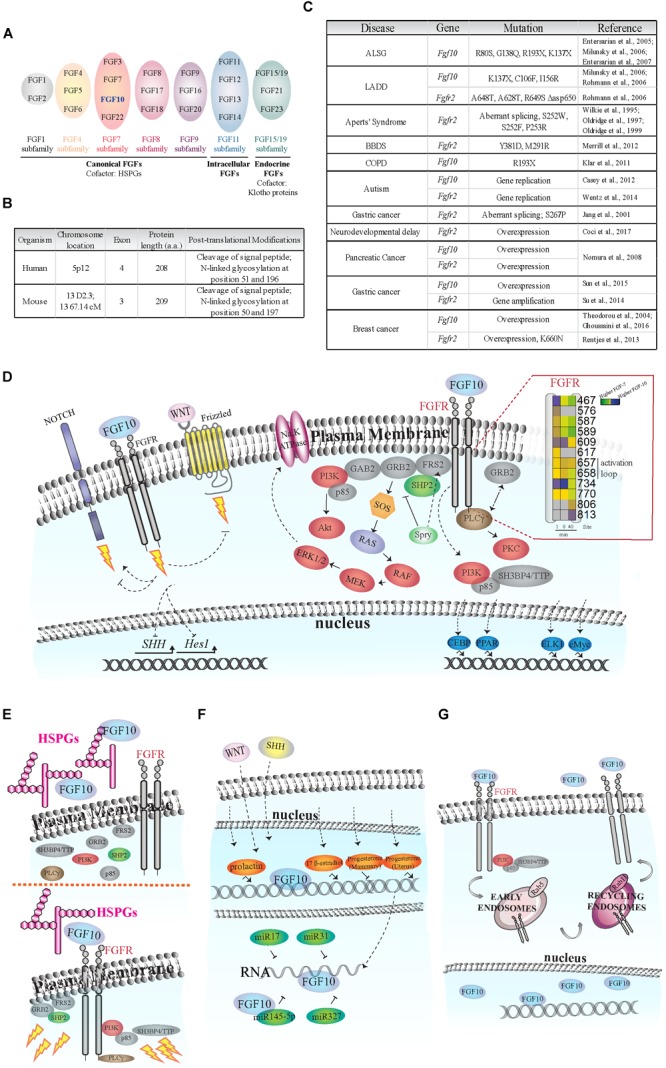
Fibroblast of Growth Factor (FGF) signaling activation and regulation. **(A)** Schematic of FGF subfamilies (see Ornitz and Itoh, 2015). FGF10 belongs to the FGF7 subfamily and is highlighted in blue. **(B)** Comparison of FGF10 gene/protein in human and mouse. The human FGF10 gene has an extra exon; and the protein length in the two species differs by one amino acid. FGF10 is secreted via the canonical ER-Golgi secretory pathway, as demonstrated by the cleavage of the signal peptide, and have two known glycosylation sites (Source: UniProt). **(C)** Known disease-causing mutations on Fgf10 and Fgfr2 genes. The rare developmental disorders Aplasia of Lacrimal and major Salivary Glands (ALSG), Lacrimo-Auricuo-Dentro-Digital (LADD) syndrome, Aperts’ Syndrome and Bent Bone Dysplasia Syndrome (BBDS) result from either the loss of key receptor-binding sites on FGF10 or mutations in the receptor kinase (TK) domains or IgII linker regions (Wilkie et al., 1995; Oldridge et al., 1997, 1999; Entesarian et al., 2005, 2007; Milunsky et al., 2006; Rohmann et al., 2006; Merrill et al., 2012). Interestingly, cases of ALSG caused by the R193X mutation also coincide with Chronic Obstructive Pulmonary Disease (COPD) (Klar et al., 2011). More recently, chromosomal translocation and duplication events at the loci of the Fgf10 and Fgfr2 genes have been associated with neurological disorders such as developmental delay and autism (Casey et al., 2012; Wentz et al., 2014). The role of single nucleotide polymorphisms and *de novo* point mutations causing oncogenic expression of Fgf10 and Fgfr2 are also becoming clearer, particularly in pancreatic, gastric, and breast cancers (Jang et al., 2001; Theodorou et al., 2004; Nomura et al., 2008; Reintjes et al., 2013; Su et al., 2014; Sun et al., 2015; Ghoussaini et al., 2016; Coci et al., 2017). **(D)** FGF10-dependent activation of FGFR intracellular tyrosine residues (Y; insert), adaptor proteins (gray), protein kinases (red), and transcription factors (blue). Insert on the right: each square represents a phosphorylated tyrosine residue (Y); the numbers correspond to phosphorylated Y residues on FGFR2b identified by proteomics; the color blue indicates higher phosphorylation at a given time point upon FGF10 stimulation of epithelial cells; modified from Francavilla et al. (2013). Dashed arrows represent FGF10-specific activation or inhibition of signaling. The lightning bolt represents the activation of signaling cascades. **(E)** Left, FGF10 bound to heparan sulfate proteoglycans (HSPGs) in the ECM does not activate FGFR and intracellular signaling. Right, FGF10 bound to HSPGs and FGFR induces the recruitment of protein adaptors to FGFR and signaling activation (represented by lightning bolts). **(F)** Schematic representation of FGF10 regulation in response to hormones (orange), miRNAs (dark green), WNT and SHH proteins. **(G)** FGF10 induces FGFR2b internalization into early endosomes and sorting to recycling endosomes and plasma membrane. FGF10 has also been found in the nucleus of certain cell types.

The Fibroblast Growth Factor 10 (*Fgf10*) gene has been identified in all examined vertebrates (Emoto et al., 1997). It belongs to the FGF7 subfamily of FGFs (Figure 1A) which was generated from a common ancestral gene during the early evolution of vertebrates and shares amino acids sequence similarities and biochemical functions (Ornitz and Itoh, 2015; Figure 1B).

Fibroblast Growth Factor 10 is a paracrine signaling growth factor of 215 amino acids with a typical signal sequence for secretion and plays an essential role during development and tissue homeostasis in adults (Itoh, 2016). *Fgf10* knockout (KO) mice die at birth with defects in multiple organ development, including the limb, lung, kidney, salivary gland and adipose tissue (Ohuchi et al., 2000). *Fgf10* gene mutations have been associated with diseases, such as aplasia of lacrimal and salivary glands (ALSG) (Entesarian et al., 2007) and lacrimo-auriculo-dento-digital (LADD) syndrome (Rohmann et al., 2006); chronic obstructive pulmonary disease (Klar et al., 2011) and certain cancer types, including breast (Theodorou et al., 2004; Ghoussaini et al., 2016), pancreatic (Nomura et al., 2008), and gastric (Sun et al., 2015) cancers (Figure 1C).

Fibroblast Growth Factor 10 activates key intracellular signaling pathways in several cell types leading to the modulation of organ branching and cell proliferation, differentiation, and migration during development; wound healing and tissue repair; maintenance of stem cells compartment; and cancer cell invasion and proliferation (Itoh, 2016). Here, we will summarize known FGF10-dependent intracellular signaling pathways and cellular responses, before focusing on how *Fgf10* expression and activity are modulated in different cellular contexts. Mechanisms underlying FGF10-dependent control of signaling specificity will be discussed and novel technologies to study the multiple roles of FGF10 will be introduced.

## FGF10-Dependent Regulators of Intracellular Signaling

### Early Signaling Players

The paracrine actions of FGF10 secreted by mesenchymal cells are mediated by the activation of epithelial FGF receptors with extracellular, transmembrane, and cytoplasmic tyrosine kinase domains (Ornitz and Itoh, 2015), and by heparan sulfate proteoglycans (HSPGs) in the extracellular matrix (ECM) (Patel et al., 2008). There are four Fibroblast Growth Factor receptor *(*Fgfr*)* genes (*Fgfr1, 2, 3, 4*). *Fgfr1–3* are alternatively spliced into “b” and “c” isoforms, which differ in their extracellular domain and ligand binding specificity and which are expressed by epithelial or mesenchymal cells, respectively (Ornitz and Itoh, 2015). FGF10 has been shown to bind and selectively activate FGFR1b and 2b (Ornitz et al., 1996; Zhang et al., 2006).

Upon FGF10 binding, FGFRs dimerize and several intracellular tyrosine (Y) residues are trans-autophosphorylated. The sequential order of tyrosine residue phosphorylation has been reported for FGFR1 (Furdui et al., 2006). The catalytic tyrosine Y653 is phosphorylated first, followed by Y583, Y463, Y585, and Y654 phosphorylation, resulting in full receptor activation. Finally, Y677 and Y766 are phosphorylated, allowing the binding of adaptor molecules to the receptor (Furdui et al., 2006). FGF10-dependent dynamic phosphorylation of FGFR2b intracellular tyrosine residues has been studied in epithelial cells using quantitative mass spectrometry (MS)-based phosphoproteomics (Francavilla et al., 2013; Figure 1D, insert). This study showed that FGF10-dependent phosphorylation of Y734 on FGFR2b (or Y730 on FGFR1) specifically induced cell migration (Francavilla et al., 2013). These findings highlight the importance of the sequence surrounding phosphorylated residues on the activated receptor for ligand-dependent activation of downstream signaling pathways.

Fibroblast Growth Factor receptors engage multiple signaling pathways via adaptor proteins (Figure 1D). FGF-regulated substrate 2 (FRS2) binds the juxtamembrane domain of FGFRs independently of receptor activation, and is phosphorylated upon ligand binding, enabling the recruitment of other scaffold proteins, such as tyrosine-protein phosphatase non-receptor type 11 (PTPN11/SHP2) and growth factor receptor-bound protein 2 (GRB2) (Ong et al., 2000). FRS2, SHP2, and GRB2 are necessary to activate the extracellular regulated kinases (ERK1/2) pathway upon FGF10 stimulation in several examined cell types (Hadari et al., 1998; Ong et al., 2000; Upadhyay et al., 2003a) and during the growth of prostate xenografts in mice (Li et al., 2018a). Furthermore, FGF10 can induce the direct recruitment of the negative ERK1/2 signaling regulator Sprouty2 to FRS2 in lung epithelial cells (Tefft et al., 2002). Sprouty2 negatively regulates FGF10-dependent trophoblast invasion (Natanson-Yaron et al., 2007), otic placode size (Mahoney Rogers et al., 2011), and lung branching (Zhao and O’Brien, 2015). GRB2 has been shown to control basal FGFR2 activation (Lin et al., 2012) by competing with the binding of 1-phosphatidylinositol 4,5-bisphosphate phosphodiesterase gamma-1 (PLCgγ) to FGFR2 (Timsah et al., 2014). PLCgγ binds to Y769 of FGFR2 (or Y766 on FGFR1) in the presence of FGF10 and is phosphorylated (Marchese et al., 2001), resulting in activation of protein kinase C (PKC) and calcium release (Figure 1D). Finally, the regulatory subunit of phosphatidylinositol 4,5-bisphosphate 3-kinase (PI3K) p85 which is known to be indirectly recruited to FGFR via the FRS2/GRB2/GAB1 complex (Ong et al., 2001), has been shown to bind to phosphorylated Y734 on FGFR2b upon FGF10 stimulation in epithelial breast cancer cells (Francavilla et al., 2013).

These results suggest context-dependent and fine-tuned modulation of downstream signaling pathways upon FGF10 binding to its receptors. FGF10 acts as a canonical FGFR ligand in recruiting the adaptor proteins FRS2, GRB2, or PLCγ to the receptor, but it can also induce the formation of cell type-specific signaling complexes (e.g., centered on p85). Either structural rearrangements of the receptor or the presence of cell type specific co-activators may explain these two different modes of FGF10 signaling initiation.

### Kinases: ERK1/2 and PI3K

Fibroblast Growth Factor receptors signal through ERK1/2 during development (Corson et al., 2003). For instance, FGF10 and ERK1/2 signaling is necessary during duct elongation of submandibular glands (Steinberg et al., 2005), epithelium tooth growth (Cho et al., 2009), and determination of vaginal epithelial cell fate in Müllerian duct epithelium (Terakawa et al., 2016). In human diseases, FGF10 is capable of stimulating the growth of endometrial carcinoma cells by activating the ERK1/2 pathway in a paracrine manner (Taniguchi et al., 2003) and is involved in the growth of ameloblastoma – an epithelial benign tumor of the odontogenic apparatus – partially signaling through ERK1/2 (Nakao et al., 2013). It has also been suggested that FGF10 has a potential therapeutic use in lung edema, as FGF10 up-regulates Na,K-ATPase activity in alveolar epithelial cells via the ERK1/2 pathway (Upadhyay et al., 2003b). Finally, the crosstalk between FGF10 and the ERK1/2 pathway has been extensively studied in cell lines, in which either manipulating the Sprouty2/FRS2 complex which controls ERK1/2 activation (Tefft et al., 2002), or inhibiting upstream activators of ERK1/2, such as MEK, resulted in decreased FGF10-induced cellular responses (Taniguchi et al., 2003; Upadhyay et al., 2003b; Francavilla et al., 2013).

The role of FGF10 in signaling regulation through PI3K and downstream kinases like protein kinase B (AKT) is less clear (Figure 1D). The PI3K/AKT pathway is important for FGF10-dependent survival of hepatoblasts during early stages of hepatogenesis (Mavila et al., 2012) and for lens development (Chaffee et al., 2016). A role for FGF10/FGFR2/PI3K in neuroprotection after cerebral ischemia has also recently been described (Chen et al., 2017).

Even though the role of other kinases in FGF10 signaling specification remains to be determined, FGF10 is a versatile growth factor that enables epithelial cell growth and migration via ERK1/2 and controls cell survival via PI3K/AKT signaling during development and in several pathological conditions.

### Transcription Factors

Fibroblast Growth Factor 10 is known to control cellular outputs through several transcription factors (Figure 1D). FGF10 plays crucial roles in adipogenesis by dynamically modulating the expression of members of the CCAAT/enhancer binding protein (C/EBP) and peroxisome proliferator activated receptor (PPAR) families of transcription factors (Sakaue et al., 2002). ELK-1 and c-MYC, but surprisingly not c-FOS, are regulated by FGF10 in endometrial carcinoma (Taniguchi et al., 2003). Finally, FGF10 controls the switch between vaginal and uterine epithelial cells fate via runt-related transcription factor 1 (RUNX1) in Müllerian duct epithelium (Terakawa et al., 2016).

As FGF10 activates both ERK1/2 and PI3K all the known transcription factors that depend on these kinases (e.g., ATF2, ELK1, FOS and FOXO, NFkB, CREB, respectively) (Yang et al., 2013; Mantamadiotis, 2017) should play a role in FGF10-dependent responses. However, it is not the case for ERK1/2-regulated activation of c-FOS (Taniguchi et al., 2003), suggesting that transcriptional regulation in response to FGF10/ERK1/2 signaling is complex and requires further investigation.

### FGF10 Crosstalk With Other Signaling Pathways

Among several other important players, we will focus on four families of proteins with a context-specific role in FGF10 signaling (Figure 1D).

Neurogenic locus notch homolog protein 1 (NOTCH1) is a receptor controlling cell signaling via several ligands and mechanisms (Bray and Gomez-Lamarca, 2018). FGF10 activates NOTCH1 signaling during pancreatic development (Hart et al., 2003; Norgaard et al., 2003) but inhibits NOTCH1-dependent regulation of the gene *Hes1* in the adult small intestine (Al Alam et al., 2015).

The functions of WNT ligands, which bind to the frizzled family of seven transmembrane receptors (Zeng et al., 2018), are modulated by FGF10 signaling during stomach (Nyeng et al., 2007) and lung (Volckaert and De Langhe, 2015) development. FGF10 also regulates Bone Morphogenetic Protein 4 (BMP4) during lung branching (Weaver et al., 2000).

The expression of *Sonic hedgehog protein (*SHH*)*, which plays crucial roles during vertebrate development (Fernandes-Silva et al., 2017), is enhanced by FGF10 during the development of limb (Yokoyama et al., 2001), prostate gland (Huang et al., 2005), and stomach (Nyeng et al., 2007).

Thus, FGF10 modulates a great variety of cellular responses during development and in pathological conditions, through both conventional and ligand-specific signaling players.

## Modulation of *FGF10* Expression and Activity

Besides known transcription factors (e.g., Tbx4/5, Isl1, Etv1/Ewsv1) (Cebra-Thomas et al., 2003; Yamamoto-Shiraishi et al., 2014; Ching et al., 2018), *Fgf10* expression and function are regulated by other factors, including HSPGs and soluble molecules (Figure 1E).

Heparan sulfate proteoglycans are a family of glycoproteins composed of a variety of heparan sulfate moieties (HS) attached to a core protein which play critical roles during organ branching and morphogenesis (Patel et al., 2017). Cleavage of HSPGs during ECM remodeling can release FGF10 from the ECM affecting epithelial cell proliferation and organ development (Figure 1E). FGF10 released from HS in the basement membrane increases salivary and lacrimal gland branching morphogenesis (Patel et al., 2008; Qu et al., 2011), whereas FGF10 binding to FGFR2b regulates the extent of the response to morphogenetics gradients (Makarenkova et al., 2009). Furthermore, the HSPG gradient pattern greatly affects FGF10 functions in the developing lung (Izvolsky et al., 2003) and during stomach morphogenesis (Huang et al., 2018). At a molecular level, FGF10 has a higher affinity for heparan compared to other FGFs (Lu et al., 1999) and it has a preference for certain patterns of sulfation and oligosaccharide length (Li et al., 2016). These unique biophysical properties of FGF10 may explain the great variety of FGF10 roles depending on cellular microenvironment, and may form the basis for the therapeutic control of FGF10 activities *in vivo*.

Fibroblast Growth Factor 10 gene expression is regulated by several hormones (Figure 1F). In mouse mammary gland 17 beta-estradiol, but not progesterone, increased the expression of *Fgf10*, whereas prolactin significantly induced *Fgf10* gene expression during pregnancy (Cui and Li, 2008). In ovine uterus, progesterone regulates *Fgf10* gene expression resulting in improved endometrial functions (Satterfield et al., 2008). These findings might be important not only to better refine FGF10 roles during development, but also to improve hormone-dependent cancer therapies. The latter possibility requires further studies to correlate hormones and FGF10 levels in human tumors, such as prostate or breast cancers.

Other important regulators of *Fgf10* gene expression during organ branching are WNT and SHH (Figure 1F). For instance, members of the WNT family are crucial for FGF10-dependent signaling in lung and limb morphogenesis (Kawakami et al., 2001; Goss et al., 2011; Volckaert et al., 2013, 2017). SHH inhibits FGF10 localized expression during lung budding (Pepicelli et al., 1998). It is worth noticing that FGF10, WNT, and SHH proteins regulate each other through the establishment of feedback loops in different cells and in a spatio-temporal regulated manner during the development of branching organs (see “FGF10 Crosstalk With Other Signaling Pathways” section above and Figure 1D), thus confirming the importance of growth factors signaling crosstalk and dynamic regulation in human development and physiology.

Fibroblast Growth Factor 10 expression can also be controlled by micro-RNAs (miRNAs), which are crucial regulators of gene expression (Dragomir and Calin, 2018; Figure 1F). The mir-17 family of miRNAs is important for FGF10/FGFR2b downstream signaling during lung bud morphogenesis (Carraro et al., 2009) and miR-31 negatively regulates expression of *Fgf10* during hair follicle growth and hair fiber formation (Mardaryev et al., 2010). More recently, it has been suggested that the miR-327/FGF10/FGFR2 signaling axis may be a therapeutic target for treatment of obesity and metabolic diseases (Fischer et al., 2017) and that the crosstalk between miR-145-5p and FGF10 expression regulates vascular smooth muscle cells proliferation and migration (Shi et al., 2018).

Although a comprehensive picture of *Fgf10* expression and activity regulation has clearly emerged, a conundrum in FGF10 signaling still remains: how is FGF10 signaling specificity controlled in different cellular contexts? The importance of subcellular localization in modulating specific aspects of FGF10 responses will be discussed in the next section.

### Subcellular Localization

The FGF10 receptor FGFR2b is internalized via clathrin-coated pits into intracellular vesicles (early endosomes) (Figure 1G), and then sorted to recycling endosomes, rather than to canonical late endosomes for degradation (Belleudi et al., 2007; Francavilla et al., 2013). Therefore, once FGFR2b is recycled back to the plasma membrane it may bind its ligands again and activate signaling in a sustained manner (Francavilla et al., 2013). Either the lack of receptor ubiquitination, which is a signal for degradation (Belleudi et al., 2007), or the recruitment of the adaptor proteins p85 and SH3BP4/TTP to phosphorylated FGFR2b (Francavilla et al., 2013) have been suggested as possible mechanisms underlying FGF10-dependent FGFR2b recycling to the plasma membrane. In either case, the sorting route of FGF10-activated receptors affects downstream signaling activation and cellular outputs, by inducing mitogenic responses in keratinocytes (Belleudi et al., 2007) or breast cancer cell migration and mouse embryonic lung branching (Francavilla et al., 2013). It would be interesting to study the endocytic route followed by FGF10 receptors in other cell types and how this affects downstream responses.

As well as in endosomes, FGF10 has been detected in the cytoplasm of cultured prostate stroma cells (Lu et al., 1999) and in the nucleus of urothelial cells (Bagai et al., 2002; Figure 1G). These findings suggest that different subcellular localization of FGF10 may underlie the specificity of FGF10 signaling in different cell types. The importance of FGF10 intracellular localization has been confirmed in studies about the molecular mechanisms underlying the LADD and ALSG human syndromes, which are characterized by mutations in the *Fgf10* gene (Rohmann et al., 2006; Entesarian et al., 2007; Figure 1C). Mutated FGF10 failed to translocate into the nucleus. This might attenuate FGF10 intracrine functions, possibly explaining the phenotype observed in LADD or ALSG patients (Mikolajczak et al., 2016).

Understanding how FGF10 regulates signaling specificity in different cell types depending on its subcellular localization and how *Fgf10* expression is modulated during organ morphogenesis and in human physiology may have therapeutic implications for cell- and growth factor-based personalized medicine.

## System Biology Approaches to Study FGF10 Signaling Specificity

**FIGURE 2:**
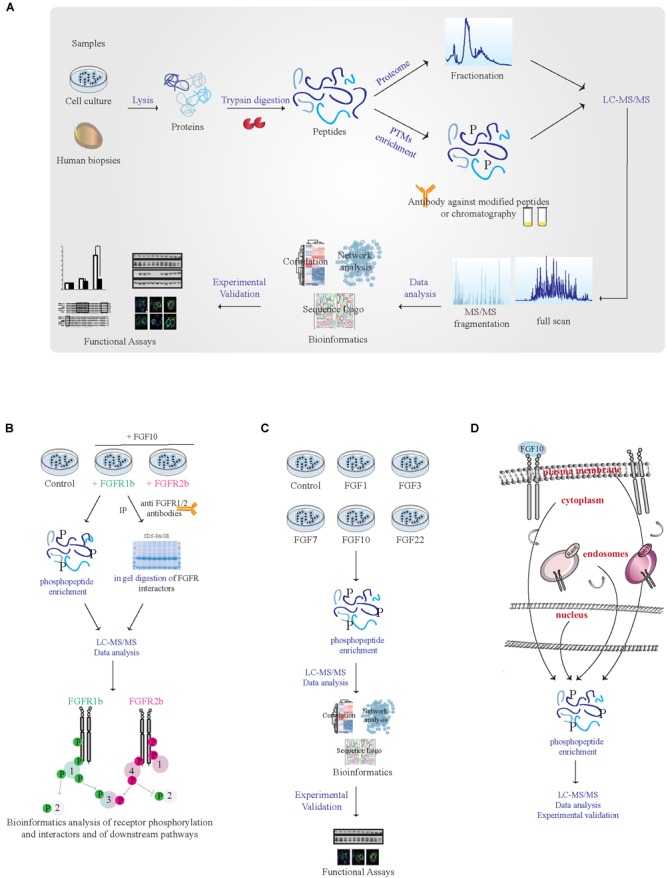
MS-based proteomic analysis of FGF10 signaling specificity. **(A)** Workflow of a typical shotgun proteomic experiment. Samples are lysed and proteins are digested into peptides. Peptides are then either fractionated to reduce sample complexity for the analysis of the whole cellular proteome or enriched for PTMs like phosphorylation using specific antibodies and chromatographic-based methods. Peptides are then separated and analyzed in the mass spectrometer. High resolution full scan and tandem MS/MS spectra are generated. Data are then analyzed by available software and bioinformatics tools before experimental validation of the most interesting hits. **(B)** Workflow of a proteomic experiment aiming at comparing FGFR1b and FGFR2b signaling in response to FGF10 stimulation. Combining phosphorylated peptide enrichment and immunoprecipitation of FGFRs followed by SDS–PAGE separation and protein in-gel digestion may result in the identification of receptor isoform-specific PTMs, protein interactors, and downstream signaling players. **(C)** Workflow of a proteomic experiment aiming at comparing signaling activation in response to different FGFs. Phosphoproteomics followed by mass spectrometry and bioinformatics will allow uncovering ligand-specific signaling cascades. **(D)** Workflow of a proteomic experiment aiming at deciphering subcellular compartment-specific signaling activation upon FGF10 stimulation. Phosphoproteomics is followed by mass spectrometry analysis and bioinformatics.

To improve our global understanding of FGF10 signaling specificity, ‘omics approaches might be useful. MS-based quantitative proteomics has become a powerful technology for investigating proteome function, composition, and post-translational modifications (PTMs) (Aebersold and Mann, 2016). In a typical shotgun proteomic workflow, proteins from tissues or cells are digested followed by peptide separation using liquid chromatography (LC) and peptide identification using tandem mass spectrometry (LC-MS/MS) (Aebersold and Mann, 2016). To identify, quantify and localize PTMs an enrichment step is introduced at the peptide level given the low stoichiometry of PTMs, such as phosphorylation (Figure 2A). In combination with functional assays phosphoproteomics has been successfully employed to study changes in intracellular signaling in tissues or perturbed cells (von Stechow et al., 2015) and one “functional proteomics” study has analyzed global FGF10 signaling in epithelial cells (Francavilla et al., 2013).

We suggest a few proteomic approaches to study FGF10 signaling specificity in an unbiased manner:

(1) Given the lack of available isoform-specific antibodies, FGF10 signaling can be compared in cells or organs expressing exclusively FGFR1b or 2b, using CRISPR-Cas9-based techniques or transgenic mice, by quantitative interactomics combined with phosphoproteomics. This will (a) increase our understanding of the relative contribution of FGFR1b and 2b isoforms to FGF10 response, as the phenotype of the FGFR2b (but not FGFR1b) KO mice resembles that of FGF10 KO mice (Ornitz and Itoh, 2015); (b) dissect which signaling players depend of FGFR1-2 activation in response to FGF10, based on recent data showing receptor-specific activation of SRC in prostate cancer (Li et al., 2018b); (c) uncover the potential role of receptor heterodimerization given the reciprocal auto-phosphorylation of FGFR1 and 2 (Bellot et al., 1991; Brewer et al., 2016); and (d) allow studying ligand-dependent receptor interactors and PTMs on the receptor (Figure 2B).

(2) System-level analysis of cellular signaling in response to different ligands for FGFR1-2b based on quantitative phosphoproteomics would reveal whether or not cellular responses are encoded by the identity of the ligand (Figure 2C). Two of the FGFR2b ligands, FGF7 and FGF10, are known to induce cell proliferation and lung cyst-like growth or cell migration and lung cell branching, respectively (Bellusci et al., 1997; Francavilla et al., 2013). However, the contribution of FGF1, 3 or 22 – which also bind to FGFR2b (Ornitz and Itoh, 2015) – has never been studied in detail. Quantitative phosphoproteomics would allow dissecting ligand-specific activation of intracellular pathways in different cell-types.

(3) MS-based organelles proteomics has been recently used to map protein subcellular localization (Itzhak et al., 2017). FGF10 signaling from different sub-cellular compartments might be dissected using a similar approach at a cellular level upon organelle enrichment followed by phosphoproteomics (Figure 2D).

## Conclusion

Fibroblast Growth Factor 10-dependent responses range from cell proliferation, migration, and invasion, to multi-organ development, cancer or genetic disease progression. Despite the enormous increase in our understanding of FGF10 signaling and regulation, gaps in our knowledge of FGF10 specificity depending on cellular and extracellular environment still exist. Systems biology approaches, including MS-based quantitative proteomics or high-content microscopy (not discussed here due to space limitations) will contribute to a full understanding of FGF10 signaling. Moving toward personalized treatments for human diseases, this knowledge will be fundamental to develop novel therapies. For instance, recombinant FGF10 or antibodies against FGF10 may be developed to modulate FGF10 signaling depending on cellular context.

## Author Contributions

All authors listed have contributed to the work and approved it for publication.

## Conflict of Interest Statement

The authors declare that the research was conducted in the absence of any commercial or financial relationships that could be construed as a potential conflict of interest.
